# Potato late blight leaf detection in complex environments

**DOI:** 10.1038/s41598-024-82272-3

**Published:** 2024-12-28

**Authors:** Jingtao Li, Jiawei Wu, Rui Liu, Guofeng Shu, Xia Liu, Kun Zhu, Changyi Wang, Tong Zhu

**Affiliations:** 1 Faculty of Information Engineering and Automation, Kunming University of Science and Technology, Kunming, 650504 China; 2 College of Plant Protection, Yunnan Agricultural University, Kunming, 650201 China; 3 School of Physics and Electrical Engineering, Liupanshui Normal University, Liupanshui, 553004 China; 4 School of Animation and Digital Arts, Communication University of China, Beijing, 100024 China

**Keywords:** Potato late blight, Disease leaf detection, Lightweight network, Attention mechanism, Plant sciences, Mathematics and computing

## Abstract

Potato late blight is a common disease affecting crops worldwide. To help detect this disease in complex environments, an improved YOLOv5 algorithm is proposed. First, ShuffleNetV2 is used as the backbone network to reduce the number of parameters and computational load, making the model more lightweight. Second, the coordinate attention mechanism is added to reduce missed detection for leaves that are overlapping, damaged, or hidden, thereby increasing detection accuracy under challenging conditions. Lastly, a bidirectional feature pyramid network is employed to fuse feature information of different scales. The study results show a significant improvement in the model’s performance. The number of parameters was reduced from 7.02 to 3.87 M, and the floating point operations dropped from 15.94 to 8.4 G. These reductions make the model lighter and more efficient. The detection speed increased by 16 %, enabling faster detection of potato late blight leaves. Additionally, the average precision improved by 3.22 %, indicating better detection accuracy. Overall, the improved model provides a robust solution for detecting potato late blight in complex environments. The study’s findings can be useful for applications and further research in controlling potato late blight in similar environments.

## Introduction

Potato, one of the most crucial food crops worldwide, faces significant losses each year due to plant diseases^1,2^. Among the various plant diseases, late blight is one of the most prevalent and serious, caused by pathogenic molds that infect potato leaves, leading to localized wilting and significantly hindering potato growth^3^. This not only affects crop yield but also results in substantial economic losses for the potato industry. Therefore, the rapid identification of late blight-infected leaves in agricultural fields are crucial for the effective prevention and management of potato diseases^4^.

Traditionally, identifying potato late blight has relied heavily on manual labor, with plant pathology experts needing to physically visit fields to identify the disease^5,6^. Unfortunately, this method has proven inefficient. However, advances in machine learning technology^7,8^ and improvements in computer hardware have led to significant progress in using image processing techniques to recognize plant diseases. Many researchers have successfully utilized clustering^9^, random forest^10^, and other methods for disease recognition, reducing both cost and labor. Nonetheless, these methods still require manual design of disease extraction features and face challenges such as limited adaptability to different situations and susceptibility to environmental factors.

With the rapid advancements in computer hardware and significant breakthroughs in deep learning, image processing techniques utilizing convolutional neural networks (CNN)^11,12^, have emerged as a prominent area of research. Numerous scholars have achieved notable success in target classification, detection, and segmentation using these neural network models. In the context of plant leaf disease recognition, the PlantVillage dataset created by Mohanty et al.^13^ has greatly facilitated research efforts, prompting many researchers to explore deep learning methodologies using this dataset. However, the images collected in laboratory settings often feature simple backgrounds and under constant lighting conditions. While some researchers have attempted to enhance the dataset through various data augmentation techniques, the effectiveness of models trained on these datasets remains constrained when applied to images captured in complex agricultural environments.

To fundamentally enhance the diversity of datasets, some researchers^14–16^, have developed plant disease leaf datasets that include complex backgrounds and have studied the recognition and detection of associated leaf diseases based on these datasets. However, these researchers often focus on addressing the low accuracy of plant leaf recognition and detection in complex environments, prioritizing improvements in model accuracy while overlooking the importance of model lightweighting. These models can achieve the desired accuracy when processing plant leaf images in complex environments. However, they are not suitable for deployment in resource-constrained edge settings because of their large number of parameters and high computational requirements.

In summary, detecting potato late blight leaves in complex environments presents several key challenges. First, the images of potato late blight leaves in publicly available datasets were primarily taken in simple laboratory settings. As a result, detection models trained on this data often perform poorly in real farmland conditions. Second, potato disease leaves exhibit significant diversity in complex environments, including variations in leaf size, capture angles, occlusions, and leaf damage. These factors make it harder to identify and detect the disease, increasing the demands on the model’s robustness and accuracy. Third, many existing detection models require substantial computational resources, which limits their use on resource-constrained edge devices. Therefore, it is essential to develop an efficient and lightweight model for detecting potato late blight leaves in complex environments.

To address the challenges mentioned above, this paper develops a YOLOv5-based lightweight model specifically designed for detecting potato late blight leaves in complex environments. This model effectively balances complexity and detection efficiency. The main contributions of this work are as follows:

- A potato late blight leaf image dataset was constructed that reflects complex environments. This dataset was created by collecting images from real farmland, along with employing multiple data augmentation techniques. This ensures the robustness of the proposed model in real scenario applications.
- The backbone network of the original YOLOv5 was enhanced by integrating ShuffleNetV2^17^, which employs a coordinate attention^18^ mechanism. This change greatly decreases the number of parameters and the computational burden of the model, while also improving its ability to extract features. Consequently, the model is better suited for use on embedded devices and reduces the rates of missed detections for potato late blight leaves in challenging environments.
- The feature fusion network of the original model was improved by incorporating bidirectional feature pyramid network^19^, which enhances the model’s ability to extract features of potato late blight leaves at different scales. This improvement also boosts the model’s generalization when dealing with potato leaves at various growth stages and from different shooting distances.

The following sections of this paper are organized as follows: Section “Related works” discusses related works on plant disease identification. Section “Materials and methods” outlines the materials used and provides a detailed description of the proposed methodology. Section “Experiments” describes the experimental environment and parameter settings. Section “Experimental results” analyzes and validates the experimental results in detail. Finally, Section “Conclusion” summarizes the main findings of this paper and offers future perspectives.

## Related works

In traditional plant image processing research, various feature extraction techniques and machine learning algorithms have been used for plant image recognition studies. For example, Nebojsa et al.^20^ improved the firefly algorithm and used it to optimize the parameters of the support vector machine algorithm, which achieved better results than the original algorithm in the task of classifying four categories of images: apples, cherries, peppers, and tomatoes. Nidhis et al.^9^ applied a clustering algorithm to categorize common rice leaf diseases, calculated the diseased area’s percentage, and thereby classified the disease severity. Govardhan et al.^10^ experimented on tomato leaf images from eight categories, comparing six machine learning methods including K-nearest neighbors, decision tree, and random forest. They found that the random forest method achieved the best results with a classification accuracy of 95%. Bukumir et al.^21^ extracted 12 features from carrots, such as length, mean diameter, and RGB color, and used a Bayesian-optimized cascade graph convolution neural network for grading. These methods based on traditional machine learning for plant image processing have shown some success, but they rely on manual feature extraction, are sensitive to environmental changes, and suffer from complex operation and poor generalization.

Compared with traditional methods, deep learning techniques are increasingly studied and utilized in plant leaf disease recognition^22^ due to their advantages like automatic feature extraction and excellent performance. For example, Eunice et al.^11^ employed various popular deep learning models for disease classification of 38 crop leaf categories in the PlantVillage dataset, finding that DenseNet-121 achieved the best performance with a mean average precision (mAP) of 99.81%. Similarly, Deng et al.^23^ constructed a classification network combining residual structure and attention mechanism, achieving 87.77% accuracy on 16 disease datasets across four plant species. Asif et al.^24^ designed a convolutional neural network model for diagnosing early blight, late blight and healthy potato leaves, attaining a maximum classification accuracy of 97%. These deep learning methods demonstrate high accuracy in classifying plant leaves and diseases, significantly aiding rapid identification and diagnosis. However, image classification alone does not fully address the complexity of diseases, such as the location of infected leaves and infection severity. Thus, employing object detection techniques is essential for a more comprehensive diagnosis of diseased leaves, providing more precise data to support disease management.

Deep learning based object detection methods are classified into two types: two-stage and one-stage. Two-stage algorithms, like Faster R-CNN, were initially dominant in target detection due to their high accuracy. However, their long training times and slow detection speeds limit their applicability in real-time industrial scenarios. In contrast, one-stage algorithms like SSD, EfficientDet, and the YOLO series have gained significant attention for their superior real-time performance and ease of use. Notably, the YOLO series has shown impressive results in both research and various industry applications. For instance, Zhong et al.^12^ developed an automated road defect detection model using YOLOv5, achieving an average accuracy of 81.6%. Qu et al.^25^ proposed a YOLOv8-LA model for underwater small target detection by improving the convolutional module and overall architecture of YOLOv8, achieving the highest mAP of 84.7% on a public dataset. Jovanovic et al.^26^ utilized both nano and small models of YOLOv8 to accomplish real-time detection of rocket bodies and engine flames, showcasing the promising applications of computer vision techniques in the field of passive rocket detection and tracking. It can be seen that the YOLO series algorithms have demonstrated effective applications across a variety of industries

In the field of plant leaf disease diagnosis, the YOLO family of algorithms has also been heavily researched and applied. For example, By incorporating an attention mechanism into YOLOv5, Zhang et al.^27^ achieved 85.59% precision and 83.70% recall in detecting grape downy mildew leaves. This advancement offers a quick and accurate deep learning approach for the automatic detection of grape leaf diseases. Zhao et al.^28^ proposed a novel loss function and integrated it into the YOLOv7 model, combining it with transfer learning. This approach achieved a mAP of 96.75% in detecting five common plant leaf diseases. Abdullah et al.^29^ enhanced the original YOLOv5 model by incorporating dilated convolution, global attention mechanism and the EIoU loss function. This modified model successfully detected healthy tomato leaves as well as nine types of diseased tomato leaves from the PlantVillage dataset. The detection results demonstrated a higher mAP of 91.40%, outperforming the original YOLOv5, as well as the YOLOv7 and YOLOv8 algorithms. Although these studies show excellent detection accuracy, the datasets were mostly collected in controlled environments with simple backgrounds and constant light. This limits the effectiveness of models trained on these datasets when applied to real farmland situations.

Some researchers have improved model performance for practical applications using images from real plant environments. Liu et al.^14^ captured images at a greenhouse tomato planting site to create a dataset of tomato brown rot with real backgrounds, achieving a detection accuracy of 94.6% using the YOLOv5 method. Yan et al.^15^ collected images of apple leaves in natural settings from the internet, creating a dataset called ALDD that includes eight typical diseased and healthy apple leaves. They achieved an average detection accuracy of 88.6% with their proposed FSM-YOLO method. Leng et al.^16^ conducted experiments using the YOLOv5 model with images of maize leaf blight taken in farmland, achieving a mAP of 87.5%. These studies are valuable for improving the practical application of leaf disease detection by building datasets in complex environments. However, these studies have primarily concentrated on improving model accuracy to tackle the challenges presented by complex environmental images, while overlooking the importance of lightweight solutions. As a result, the models may be too complex to deploy effectively on resource-constrained embedded devices.

In conclusion, recent studies mostly focus on datasets from controlled environments, overlooking the complexity of real farmland environments, which limits the model’s generalization. Several studies have achieved high detection accuracy using datasets from real farmland environments. However, the complexity of these models presents challenges when deploying them on edge devices with limited computational resources. The work in this paper should focus on improving detection accuracy in complex settings, while also enhancing model efficiency and lightweight design to meet computational limits and real-time needs in practical applications.

## Materials and methods

### Data collection and processing

**Figure 1:**
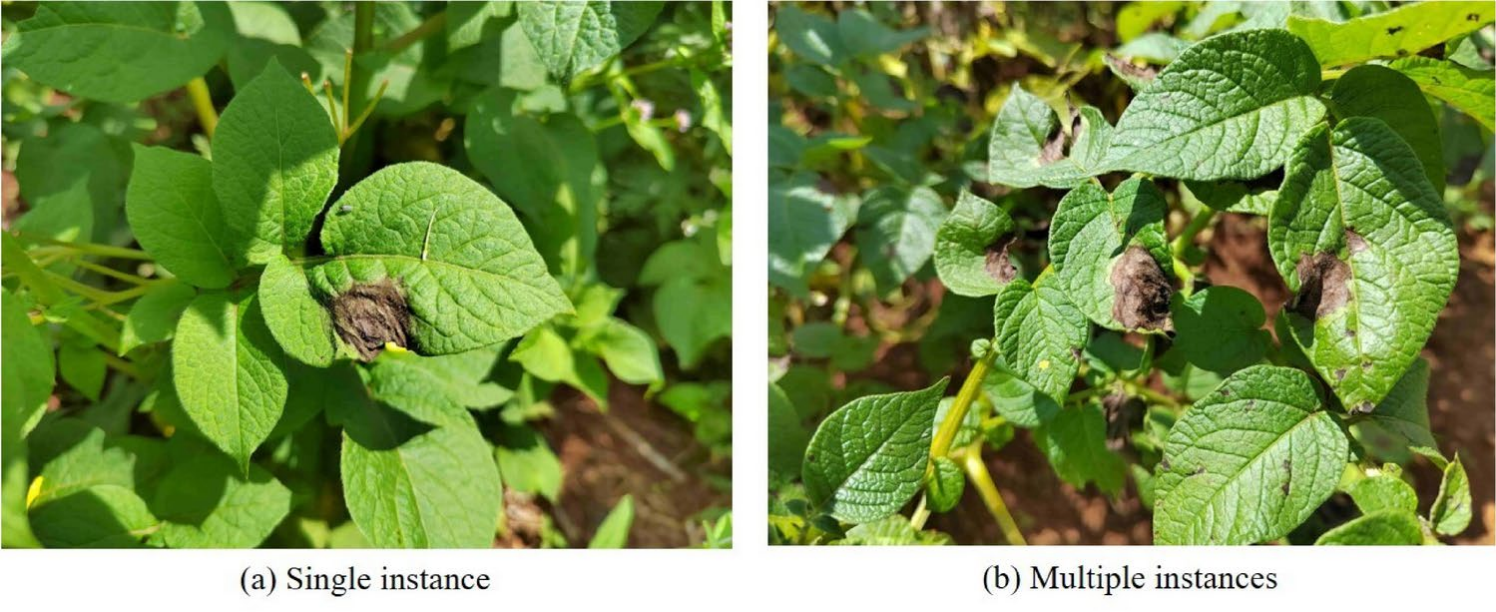
Potato late blight leaves in complex environments.

To create a dataset specifically for potato late blight leaf detection in complex environments, a total of 2592 images were collected from modernized potato planting bases in Yunnan Province and various internet sources. Due to limited storage space, the size of these images was reduced from 7.72 GB to 342 MB using the compression tool docosmall. Some of the processed pictures are shown in Fig. 1.

The collected images were then labeled using LabelImg, resulting in 27,540 instances of late blight leaves. To expand the dataset, random rotation, mirroring, and scaling operations were applied to the original images. Additionally, brightness adjustments, color variations, and noise were introduced to further augment the dataset. These processes produced a total of 20,736 images. After manually filtering out poor-quality images, a final dataset of 19,950 images with 215,856 labeled instances was obtained.

The dataset was then divided into three sets: training, validation, and testing, in a ratio of 7:2:1. Care was taken to ensure that the average number of annotations per image was similar across all three sets, guaranteeing balanced representation. During the training process, the Mosaic data augmentation method included in YOLOv5 was disabled. This decision was made to prevent any potential negative impact from overlapping data augmentation techniques and to conserve computational resources.

### Improved YOLOv5-based detection

YOLOv5 is a algorithm derived from the real-time, single-stage target detection algorithm YOLO (You Only Look Once). In October 2021, Ultralytics introduced the sixth iteration of YOLOv5, which is comprised of four versions: YOLOv5s, YOLOv5m, YOLOv5l, and YOLOv5x. The primary distinction between these versions lies in the depth and width of the network, which influences the quantity of parameters and the training duration of the model. Over time, newer YOLO versions like YOLOv6, YOLOv7, and YOLOv8 have been proposed. However, YOLOv5 still has a strong accumulation of community technologies, which provide a large number of solutions for development and deployment. It will take time for new versions to gain similar support. Additionally, YOLOv5, due to its earlier introduction and proven advantages, has been successfully applied in various industrial scenarios, demonstrating mature experience and positive outcomes. In contrast, while YOLOv8 has shown superior performance on standard test datasets, its application across diverse environments in different scenarios still requires extensive validation. Therefore, this paper chooses YOLOv5 as the foundational research framework for detecting potato late blight in complex environments, aiming for efficient development and stable deployment.

**Figure 2:**
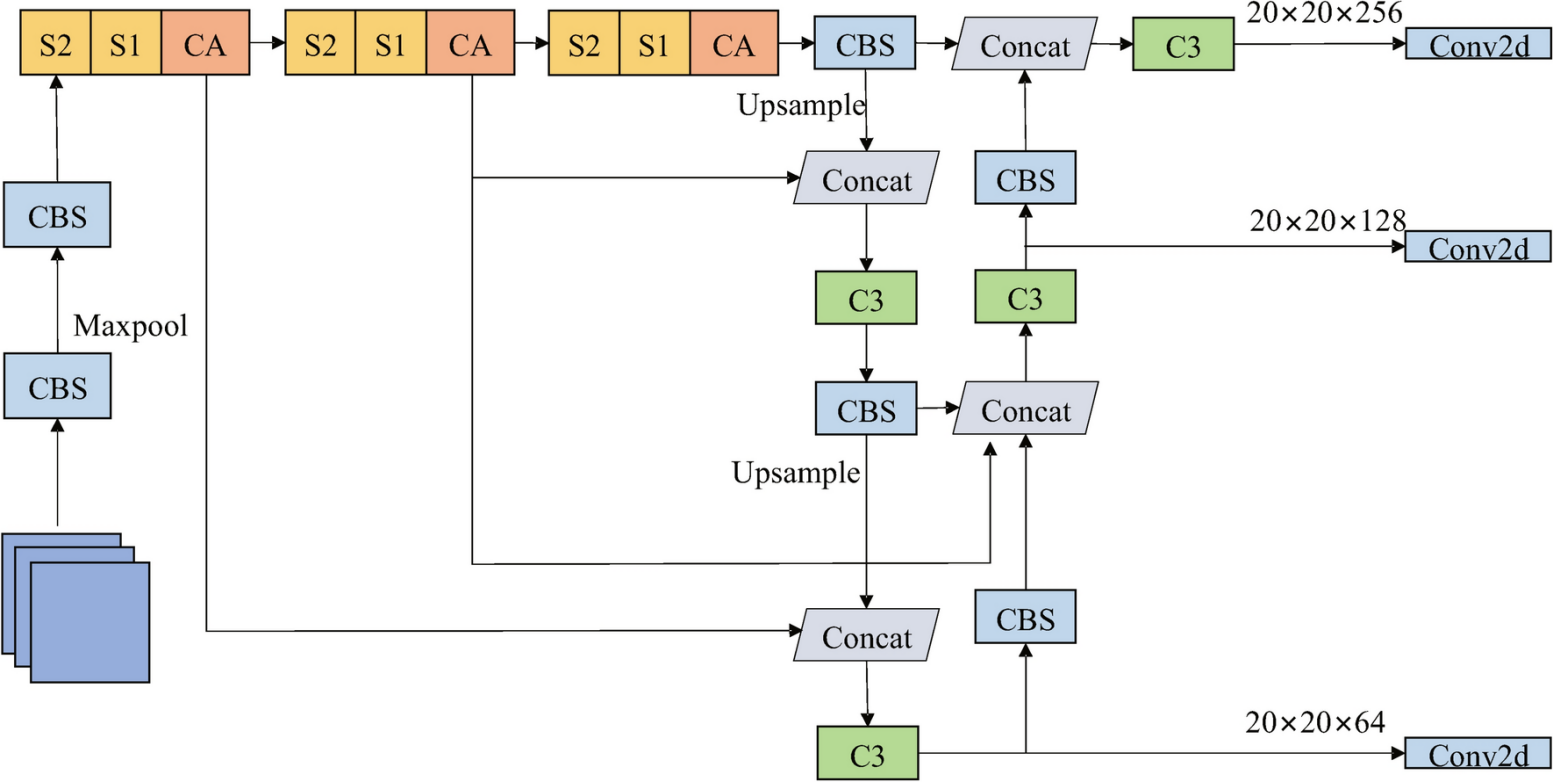
Improved YOLOv5 network structure.

To adapt the original YOLOv5 model to detect potato late blight leaves in complex farm environments, this study incorporates several key improvements. Firstly, ShuffleNetV2 is employed as the backbone network to fulfill the lightweight requirements. Secondly, a coordinate attention mechanism is integrated into the backbone to enhance detection accuracy and reduce false negatives. Thirdly, BiFPN is used to merge features of different scales, thereby enhancing the model’s feature expression capability and improving the detection of small targets. The improved YOLOv5 is illustrated in Fig. 2.

#### Lightweight backbone network

In large potato fields, implementing disease detection models on edge devices such as UAVs (Unmanned Aerial Vehicles) offers a practical solution^30^ for automated disease detection. However, the limited storage and performance of these edge devices make traditional detection models unsuitable^31^. Therefore, this paper uses ShuffleNetV2 to lighten the YOLOv5 backbone network, making it more suitable for edge device deployment.

**Figure 3:**
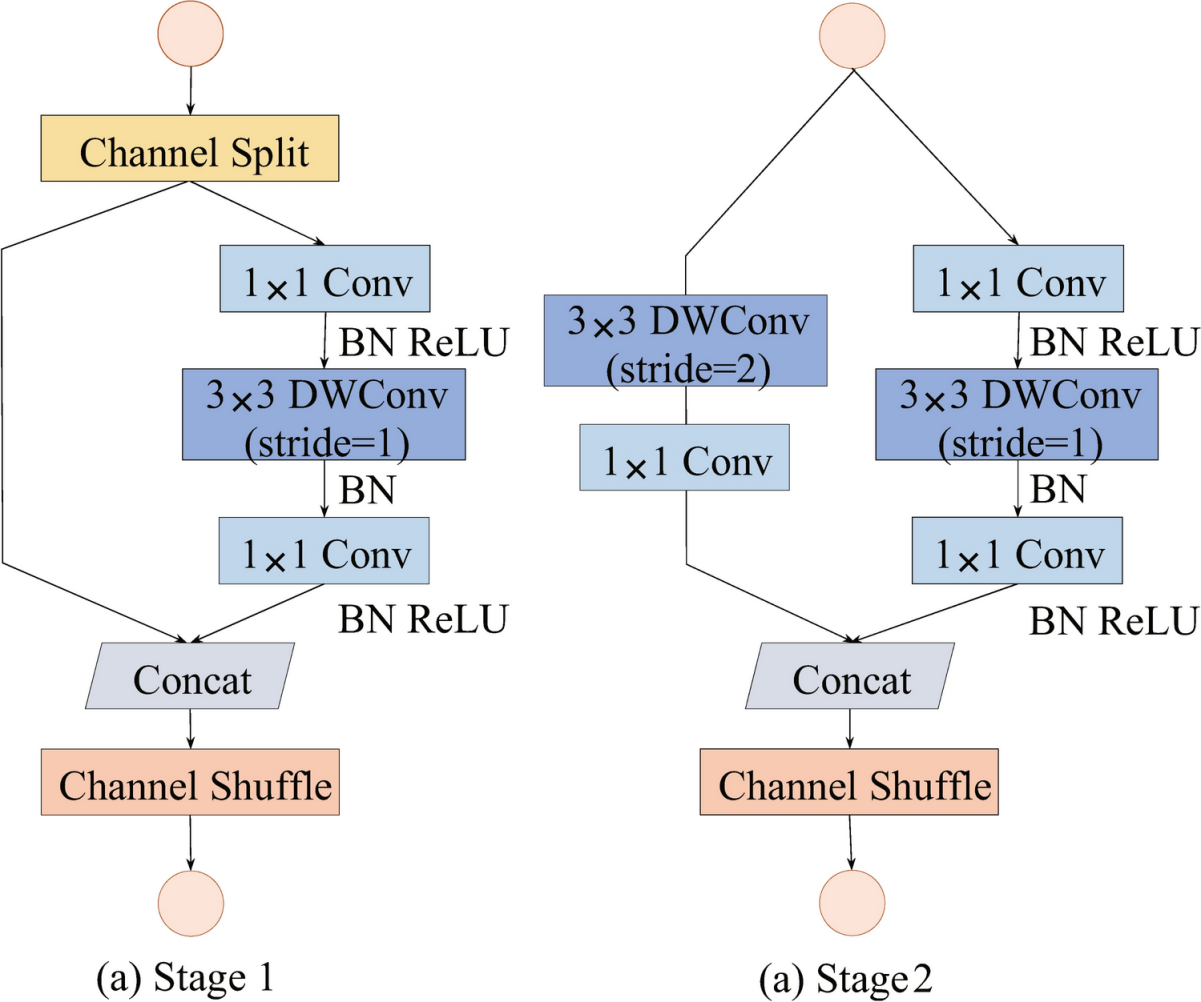
Two unit structures for ShuffleNetV2.

ShuffleNetV2 is a lightweight network architecture designed for edge devices. Fig. 3 illustrates two main unit structures of ShuffleNetV2. Stage 1 is the basic unit of ShuffleNetV2. It starts by dividing the input feature map into two segments via channel splitting. One segment is processed through three convolution operations, while the other remains unprocessed. These segments are then concatenated, followed by a channel shuffle operation to facilitate information exchange among channels. Stage 2 is the downsampling unit and differs from Stage 1 by not splitting the input feature map. Instead, it processes the input through deep convolution operations on two separate routes. The resulting feature maps are concatenated and subjected to a channel shuffle operation, which doubles the number of channels in the final output. This stage is designed to increase the network’s capacity without a significant increase in computational complexity.

#### Coordinate attention

In image processing tasks, attention mechanisms^32^ assigns weights to different features of the input data like channels and locations, enabling the model to focus on key areas and thus improve its ability to select and weight features. This dynamic weight allocation not only enhances the model’s accuracy in complex scenarios but also strengthens its robustness, allowing it to better adapt to various environments. In the task of detecting potato late blight in complex environments, factors such as occlusion, complex backgrounds, and lighting changes can interfere with the model’s judgment. Attention mechanisms can automatically adjust the importance of each position or channel, highlighting relevant areas for disease leaf recognition and localization while ignoring irrelevant backgrounds. This process enhances the model’s ability to extract features related to diseased leaves^33^ and ultimately improves the accuracy of disease leaf detection.

To address the challenges of detecting potato late blight in complex environments, this paper introduces the coordinate attention (CA) mechanism into the YOLOv5 network architecture. Coordinate attention is a hybrid attention model that integrates position information into channel attention and encodes it bidirectionally. This approach enables the model to capture both channel relationships and long-distance dependencies more effectively. The CA mechanism consists of two main steps: coordinate information embedding and coordinate attention generation. Specifically, for a feature map *x* with an input size of $C{\times }H{\times }W$, a pooling kernel of size (*H*,1) is used to encode along the horizontal direction. This operation results an output $z^h$ with the number of channels *C*, height *H*, and width 1. The output $z^h$ for the *h*-th row of the *c*-th channel can be described as follows:

1 $$\begin{aligned} z_c^h\left( h\right) =\frac{1}{W}\sum _{0\le i<W}{x_c(h,i)} \end{aligned}$$

In the above equation, $x_c(h, i)$ represents the value of the feature map *x* at the *c*-th channel, located in the *h*-th row and *i*-th column. In a similar manner, the output $z^w$ for the *w*-th column of the *c*-th channel in the vertically encoded result can be defined as follows:

2 $$\begin{aligned} z_c^w\left( w\right) =\frac{1}{H}\sum _{0\le j<H}{x_c\left( j,w\right) } \end{aligned}$$

Likewise, $x_c(j, w)$ has the same meaning as $x_c(h, i)$ in Eq. (1). The two output feature maps mentioned above are concatenated into a new feature map of size $C{\times }1{\times }$(*H*+*W*). This concatenated feature is then downsampled using a 1$\times$1 convolution, denoted as $F_1$. Finally, a non-linear activation function $\delta$ is applied to obtain the final output, *f*. This can be represented as follows:

3 $$\begin{aligned} f=\delta (F_1([z^h,z^w])) \end{aligned}$$

where [,] denotes the concatenation operation along the spatial dimension. Following the above steps, the output *f* is split into two components $f^h$ and $f^w$. These two feature maps are then transformed into two new feature maps, respectively, using two separate 1$\times$1 convolutional kernels, $F_h$ and $F_w$. At this point, the horizontal and vertical attention weights $g^w$ and $g^h$ are obtained. This can be described as:

4 $$\begin{aligned} g^h&=\sigma (F_h(f^h))\end{aligned}$$

5 $$\begin{aligned} g^w&=\sigma (F_w(f^w)) \end{aligned}$$

The $\sigma$ in the above is the sigmoid activation function. In the final step, the feature information of $g^h$ and $g^w$ are fused and then multiplied with the original feature map *x* to yield the final coordinate attention module, denoted as *y*. The weight value for the *c*-th channel in this module is defined as:

6 $$\begin{aligned} y_c\left( i,j\right) =x_c\left( i,j\right) \times g_c^h\left( i\right) \times g_c^w\left( j\right) \end{aligned}$$

**Figure 4:**
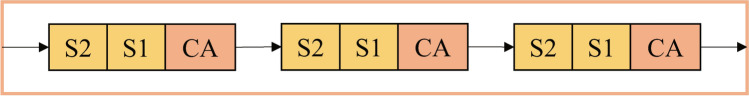
Backbone with added CAs.

As mentioned in the above equation, $g_c^h\left( i\right)$ represents the attention value for the *c*-th channel at *i*-th row, while $g_c^w\left( j\right)$ denotes the attention value for the *c*-th channel at *j*-th column. By consecutively multiplying them with the value $x_c\left( i,j\right)$ at the i-th row and j-th column of the c-th channel in the feature map *x*, the final weight $y_c\left( i,j\right)$, incorporating the coordinate attention mechanism, is obtained. In this study, three coordinate attention modules are incorporated after each stage 1 of ShuffleNetV2. The placement of these additional coordinate attention modules is illustrated in Fig. 4.

#### Bidirectional feature pyramid network

In practical applications, such as detecting potato leaves in a field, the size of potato leaves can vary with the growth stages. Moreover, leaves from the same plant may differ in size, and their apparent size can also change depending on the shooting distance. Therefore, fusing features from different scales is crucial to improve the model’s ability to detect potato late blight leaves of various sizes.

The Feature Pyramid Network^34^ (FPN) enhances by merging feature information from feature maps of various scales within the backbone network. This process allows larger-scale feature maps to retain higher dimensional feature information while compensating for any potential loss during the downsampling of smaller-scale feature maps.

**Figure 5:**
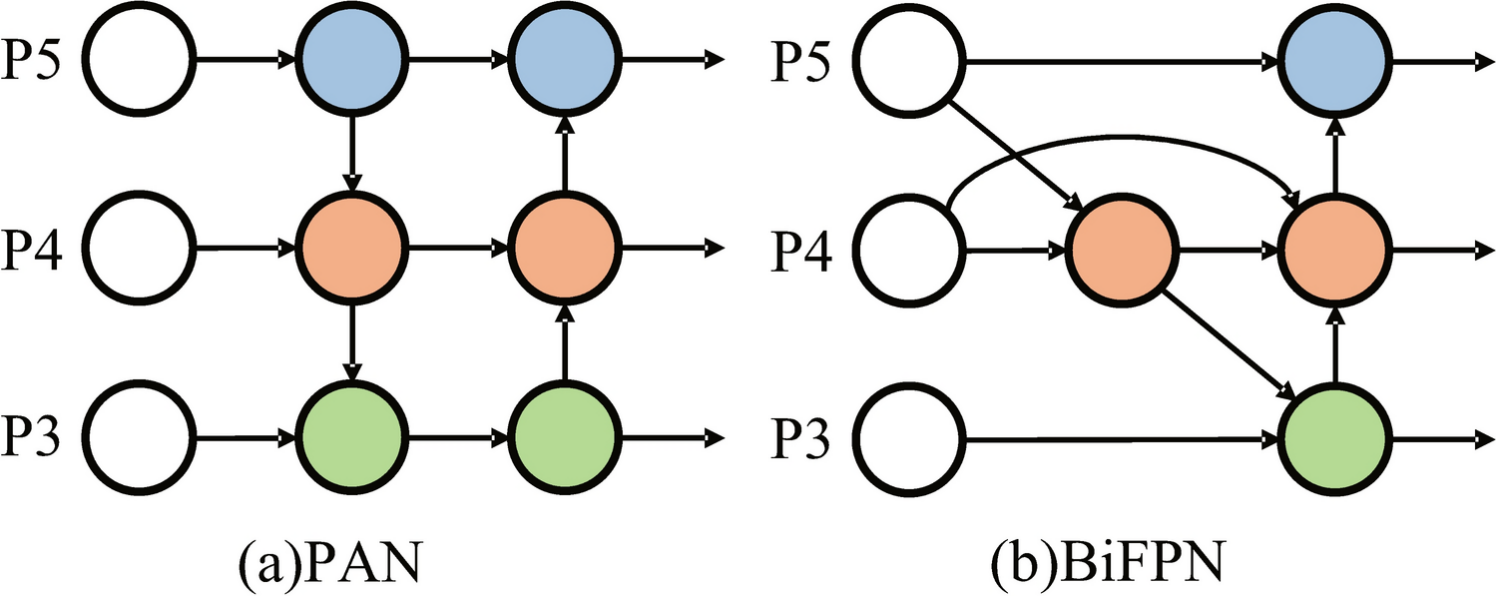
PAN and BiFPN.

In the original YOLOv5, a Path Aggregation Network (PAN) based on improved FPN is used as the feature fusion network. This network enables bidirectional fusion of features across different scales. However, PAN simply integrates features through a cascading structure without considering the weighted contributions of feature maps at different scales during fusion, which leads to high computational complexity and less efficient. To address these issues, Tan et al.^19^ proposed the Bidirectional Feature Pyramid Network (BiFPN) in EfficientDet. BiFPN employs a weighted feature fusion technique where different scales of features are combined using learnable weights. This adaptive mechanism helps in effectively merging features of varying resolutions and importance, leading to better feature representation. Compared to PAN, BiFPN performs feature fusion more efficiently, significantly enhancing the model’s performance. The structural comparison of these two feature fusion networks is illustrated Fig. 5.

## Experiments

### Experiment settings

In the experiments conducted in this paper, consistency across all hardware and software environments was ensured to maintain the comparability and reliability of the results. The hardware configuration includes a CPU setup of 6 $\times$ Xeon E5-2678 v3 and an NVIDIA GeForce RTX 2080 Ti GPU with 16GB of memory. On the software side, Ubuntu 18.04 was used as the operating system, Python 3.8 as the programming language, PyTorch 1.10 as the deep learning framework, and CUDA 11.3 for GPU acceleration.

The input size for all training and testing images was standardized to 640 pixels to ensure consistency in image scale across different model configurations. During training, a batch size of 16 and 300 epochs were utilized. The initial learning rate was set at 0.01 to facilitate rapid convergence in the early stages of training, while a cyclic learning rate of 0.002 was employed to dynamically adjust according to changes in the loss curve. Stochastic gradient descent (SGD) was employed for backpropagation, with a momentum of 0.94 and a weight decay of 0.0005. Additionally, a confidence threshold of 0.5, and a non-maximum suppression threshold was established at 0.3.

### Evaluation metrics

In this experiment, the evaluation metrics were primarily chosen based on the requirements for deploying the detection model in real-world agricultural settings. The main focus was on the model’s detection performance and its lightweight nature. To evaluate the detection performance of the model, Precision (P), Recall (R), and Average Precision (AP) were used as the evaluation metrics. The three evaluation metrics are formulated as:

7 $$\begin{aligned} P&=\frac{TP}{TP+FP}\times 100\%\end{aligned}$$

8 $$\begin{aligned} R&=\frac{TP}{TP+FN}\times 100\%\end{aligned}$$

9 $$\begin{aligned} AP&=\int _{0}^{1}P\left( R\right) dR \end{aligned}$$

In the formulas above, TP (True Positive) refers to the number of positive samples correctly identified as positive. FP (False Positive) denotes the number of negative samples incorrectly identified as positive. FN (False Negative) represents the number of positive samples incorrectly identified as negative. Furthermore, this study employs the number of parameters, floating point operations (FLOPs), and frames per second (FPS) as evaluation metrics for model lightweighting. These metrics are critical for assessing model efficiency, particularly given the limited computational resources in practical applications and the necessity to measure the degree of model lightweighting.

## Experimental results

### Comparison of backbone networks

To demonstrate the feasibility of the lightweight improvement method proposed in this paper, this section employs CSPDarknet53, MobileNetV3^35^, and ShuffleNetV2 as the respective backbone networks. The experiments compare the differences in AP, parameter count, and FLOPs among these different backbone network models. Table 1 presents the model detection performance of YOLOv5 under different backbone networks. The results indicate that both MobileNetV3 and ShuffleNetV2 significantly reduce the parameters and FLOPs compared to the original YOLOv5 model with CSPDarknet53 as the backbone network. Notably, the YOLOv5 model with MobileNetV3 as the backbone network exhibits the most pronounced lightweighting effect, with a reduction of 3.48M parameters and FLOPs decreasing from 15.9$\times 10^{9}$ to 6.3$\times 10^{9}$. However, this comes at the cost of a nearly 6% decrease in AP. Considering the objective of lightweighting the backbone network while minimizing accuracy loss, this paper selects ShuffleNetV2 as the lightweight backbone network due to its superior overall performance. Although the YOLOv5 model with ShuffleNetV2 as the backbone network has slightly higher parameters and FLOPs compared to MobileNetV3, its AP is 2.66% higher. This choice aligns better with the requirements of potato late blight leaf detection in complex environments addressed in this study.

**Table 1:** Comparison of different backbone networks.

Model	Backbone	AP@0.5 / %	Params /$10^{6}$	FLOPs / $10^{9}$
YOLOv5	CSPDarknet	74.43	7.02	15.94
	ShuffleNetV2	72.87	3.76	8.16
	MobileNetV3	70.21	3.54	6.34

### Comparison of attention mechanisms

**Figure 6:**
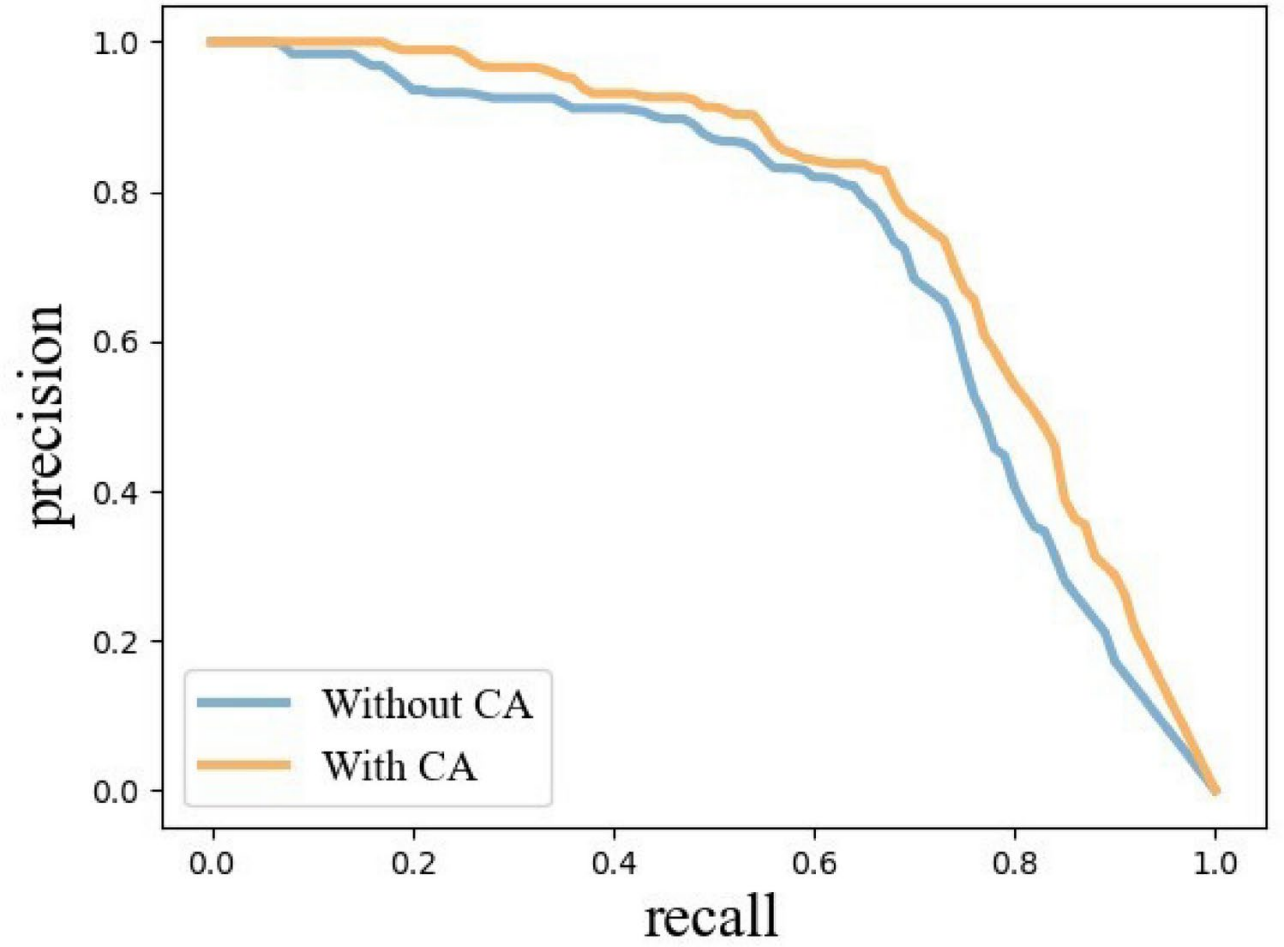
Comparison of P-R curves with and without CA.

In this section, the introduction of attention mechanisms into the lightweight backbone network is discussed. The attention mechanisms incorporated include SE^36^ (Squeeze-and-Excitation networks), CBAM^37^ (Convolutional Block Attention Module), and CA. Experiments are conducted to compare their potential enhancements on the detection performance of the YOLOv5-ShuffleNetV2 network. The results of these experiments are presented in Fig. 6 and Table 2.

**Table 2:** Comparison of different backbone networks.

Attention mechanism	AP@0.5 / %	Precision/ %	Recall / %	Params / $10^{6}$
–	72.87	75.42	71.38	3.76
SE	75.16	77.25	74.25	3.81
CBAM	75.91	76.80	74.84	3.81
CA	76.51	77.42	76.14	3.80

Table 2 provides a detailed comparison of various attention mechanisms on the model’s performance metrics, including AP, precision, recall, and parameter count. Without introducing any attention mechanism, the network exhibits significantly lower recall compared to precision, leading to frequent misses in detecting potato late blight leaves in complex environments. When the SE mechanism is introduced, both precision and recall see improvements, with precision increasing by 1.83% and recall by 2.87 %. The CBAM further enhances AP and recall,although its precision is slightly lower than that achieved with SE. The addition of the CA leads to the greatest improvement in AP, precision, and recall, showing AP increasing by 3.64%, precision by 1.93%, and recall by 4.76%, compared to the original model without any attention mechanism. As illustrated in Fig. 6, the P-R curves also demonstrates improved precision and recall upon incorporating CA. Additionally, the CA attention mechanism adds the fewest parameters, aligning well with this study’s goal for a lightweight model. In conclusion, the model incorporating CA most effectively meets the experimental expectations by significantly enhancing the original network’s low precision and recall for detecting potato late blight leaves, all while minimally increasing the parameter count.

### Ablation experiments

**Figure 7:**
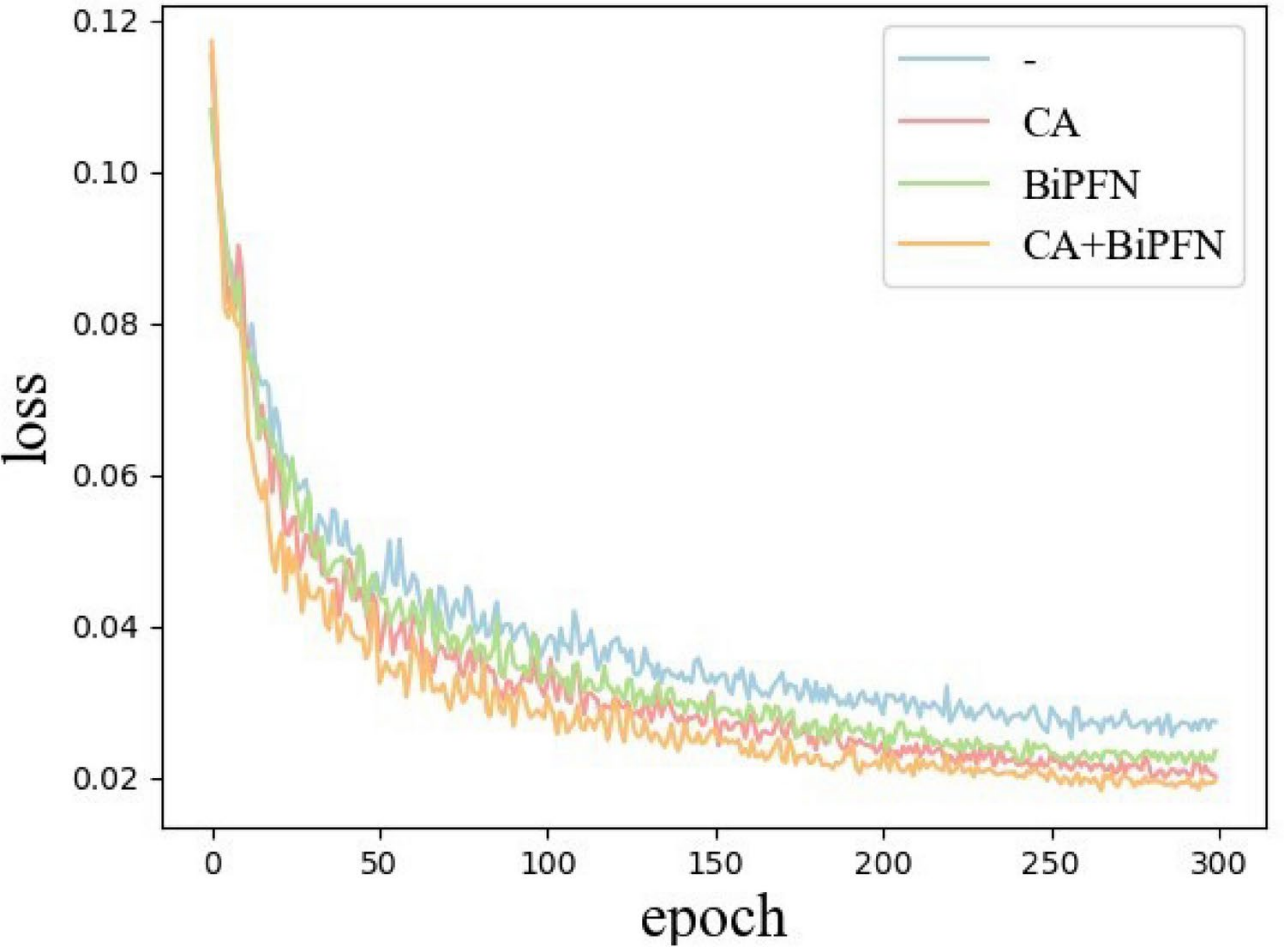
Comparison of loss with different improvement modules.

To validate that the enhancement strategies proposed in this paper effectively improve the detection of potato late blight leaves in complex environments. Based on the YOLOv5-ShuffleNetV2, this section includes CA and BiFPN into the network as independent validation modules for ablation experimental analysis. The experimental results are presented in Table 3 and Fig. 7. In the table, a “$\checkmark$” means the module is used, while a “—” means it is not.

**Table 3:** Results of ablation experiments.

Modules		AP@0.5 / %	Params/$10^{6}$	FLOPs /$10^{6}$	Box loss
CA	BiFPN				
—	—	72.87	3.76	8.16	0.0270
$\checkmark$	—	76.51	3.80	8.22	0.0208
—	$\checkmark$	75.40	3.83	8.37	0.0226
$\checkmark$	$\checkmark$	77.65	3.87	8.43	0.0192

As shown in Table 3, the AP of the original lightweight network improves after adding either CA or BiFPN individually, with CA showing an additional 1.11% increase compared to BiFPN. When both modules are added simultaneously, the model’s detection performance sees further improvement, achieving a 4.78% increase in AP with only a 0.11M rise in parameters, maintaining the model’s lightweight nature. Fig 7 illustrates the comparison of loss values during model training with different improvement modules. The results show that the model converges the fastest and achieves the lowest final loss value when both modules are implemented together. This outcome aligns with the study’s expectations for enhancing potato late blight leaf detection in complex environments, effectively accomplishing the detection task in such scenarios.

### Comparison of different size models

This section compares the proposed method with YOLOv5 and YOLOv8 to evaluate the balance between detection accuracy, number of parameters, and computational complexity for different model sizes. The goal is to choose the best potato late blight leaf detection model for the scenarios discussed in this paper.

Table 4 shows that larger models improve detection accuracy for all models, especially for YOLOv5 and YOLOv8, highlighting the benefits of larger models. However, these improvements come with more parameters and higher computational needs, which may limit the deployment of the model in resource-constrained devices. YOLOv8 outperforms YOLOv5 in detection accuracy for same sizes, although models of the same size generally have more parameters and require more computation, these enhancements are manageable on devices with sufficient resources.

The proposed method achieves a lightweight model comparable to the original YOLOv5 while also improving average precision across all size configurations. Specifically, it enhances AP by 3.22% for small size, 1.51% for medium size, and 0.86% for large size. This suggests that the proposed improvement strategy is especially effective for smaller YOLOv5 models, offering a more optimized solution for lightweight models used in detecting potato late blight on leaves. Compared to YOLOv8, the proposed model maintains higher accuracy in small size while being more lightweight. Although its detection accuracy for medium and large configurations is slightly lower than that of YOLOv8, the proposed model clearly outperforms in terms of reduced parameter count and computational load. Overall, the proposed method optimally balances detection accuracy, parameter count, and computational complexity in the small size model. It reduces parameters and computation while maintaining high accuracy, making it more suitable for applications in resource-limited edge devices.

**Table 4:** Comparison of different size models.

Model	Size	AP@0.5 / %	Params / $10^{6}$	FLOPs / $10^{9}$
YOLOv5	S	74.43	7.02	15.94
	M	76.72	20.87	48.22
	L	77.86	46.13	108.23
YOLOv8	S	76.11	11.17	28.81
	M	78.46	25.90	79.32
	L	79.58	43.69	165.74
Proposed method	S	77.65	3.87	8.43
	M	78.23	10.06	21.58
	L	78.72	20.34	43.20

### Comparison with other methods

In this section, the proposed method is rigorously compared with other lightweight algorithms to substantiate its superior efficacy in detecting potato late blight leaves in complex environments. The experiments meticulously control variables and utilize the same dataset to ensure uniform experimental conditions. The outcomes of the experiment are presented in Table 5.

**Table 5:** Comparison of different lightweight networks.

Modules	AP@0.5 / %	Params/$10^{6}$	FLOPs /$10^{6}$	FPS
YOLOv5s	74.43	7.02	15.94	44
YOLOv3-tiny	69.31	8.67	12.99	**87**
YOLOv5-ghost^38^	73.89	**3.46**	**7.21**	48
Proposed method	**77.65**	3.87	8.43	51

**Figure 8:**
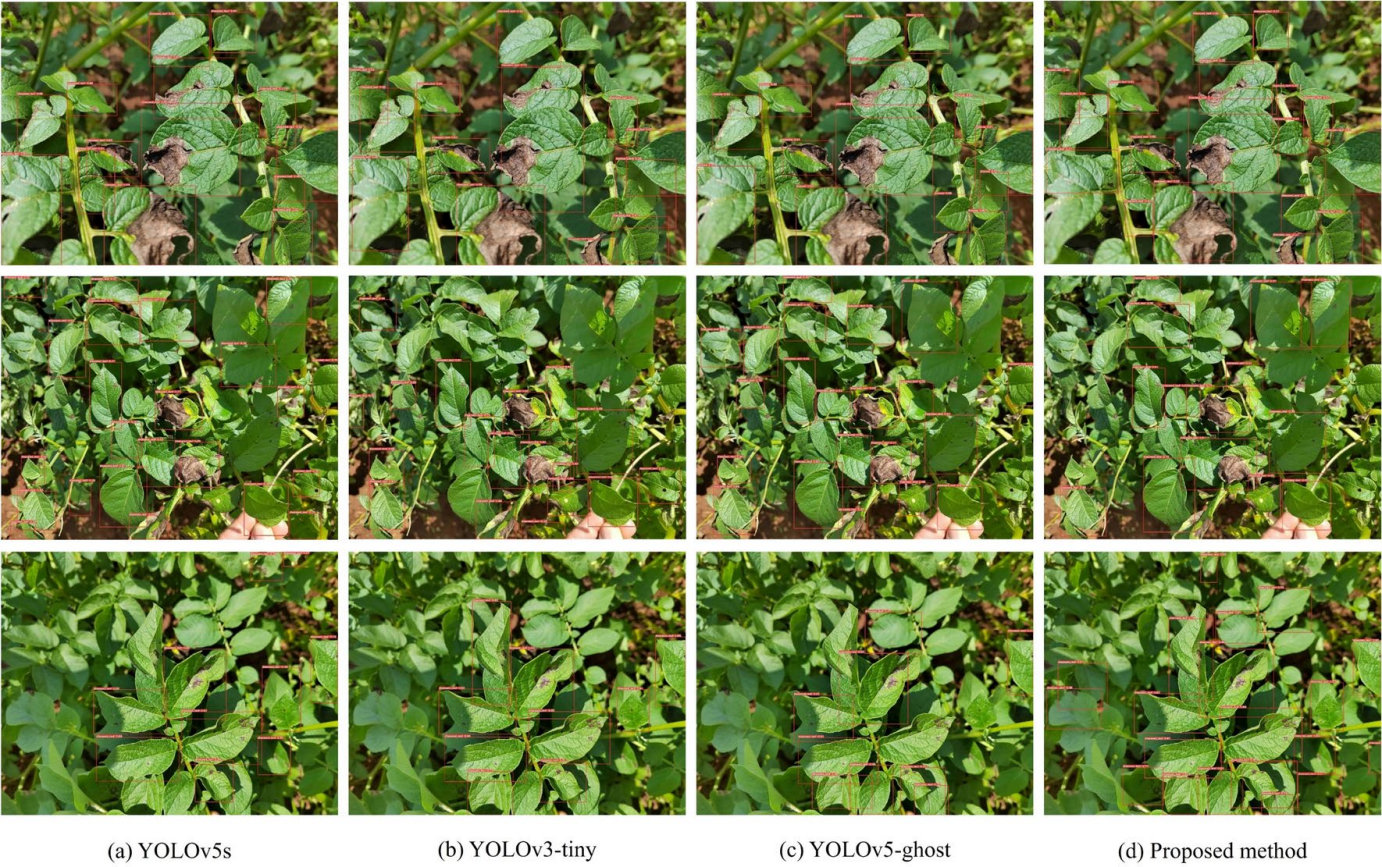
Detection results of different lightweight networks.

Based on the comparison data in Table 5, it is evident that the YOLOv5s algorithm has the highest FLOPs and the slowest detection speed. In contrast, the YOLOv3-tiny algorithm achieves the fastest detection speed at 87 FPS but has the lowest AP. The YOLOv5-ghost algorithm, while being the lightest, has lower AP and FPS compared to the method proposed in this paper. The proposed method exhibits the highest AP, with parameters and FLOPs slightly higher than YOLOv5-ghost but significantly lower than other methods. It achieves an AP of 77.65% and a detection speed of 51 FPS, both surpassing YOLOv5s and YOLOv5-ghost. In conjunction with Fig. 8, the method proposed in this paper achieves an optimal balance among detection accuracy, model complexity, and detection speed, resulting in superior overall performance. It proves to be an effective approach for detecting late blight on potato leaves in complex environments.

## Conclusion

In this work, an enhanced YOLOv5 model designed for deployment in farmland settings was introduced, with the aim of detecting potato late blight leaves in complex environments. A specialized dataset was created, followed by extensive experiments. The main work is in the following aspects: Firstly, various backbone networks were compared, and ShuffleNetV2 was selected as the lightweight backbone. This choice significantly reduces the model’s parameters and computation while maintaining detection accuracy, facilitating deployment in resource-constrained embedded devices. Secondly, building on lightweighting, the study examined various attention mechanisms’ effects on model performance. Results indicated that introducing the CA mechanism significantly improved the model’s AP value and recall rate, effectively reducing missed detections of potato late blight leaves in the original model. Next, ablation experiments verified that combining CA and BiFPN further enhances the model’s performance while preserving its lightweight feature. Finally, the proposed method is compared with other methods. Experiments show that the proposed improvement strategy achieved the highest increase in AP at small size, reaching a value of 3.22%, while also offering the most lightweight solution. Compared to other lightweight detection methods, it excels in detection accuracy and speed, achieving an optimal balance in overall performance. In summary, this paper’s key contribution is the development of an efficient and lightweight method for detecting potato late blight in complex environments. This method balances detection accuracy, model complexity, and speed, offering effective technical support for diagnosing late blight in real potato farmland scenarios.

The proposed method accurately and quickly detects potato late blight leaves in complex environments but has limitations. It can identify diseased leaves but cannot measure how severe the disease is. This is important in potato farming because different severity levels need different treatments to keep plants healthy and productive. The inability of the current method to assess severity could potentially reduce control effectiveness and lead to resource waste or unnecessary chemical use. Therefore, future research should include area calculations for leaves and diseases based on the proposed detection model. Additionally, it should precisely assess potato late blight severity through diseased area percentage. This severity grading will offer more effective decision support and a comprehensive diagnostic information for the control of potato late blight.

## Data Availability

The datasets generated and analyzed during the current study are available from the corresponding author on reasonable request.
